# The comet initiative

**DOI:** 10.1186/1745-6215-14-S1-O65

**Published:** 2013-11-29

**Authors:** Paula Williamson

**Affiliations:** 1 University of Liverpool, Liverpool, UK

## 

There is growing recognition that insufficient attention is paid to the outcomes measured and reported in clinical trials. Selection of outcomes is crucial to trials designed to compare the effects of different interventions. For the findings to influence policy and practice, the chosen outcomes need to be relevant to patients and the public, healthcare professionals and others making decisions about health care.

Trials in a specific condition often report different outcomes, or address the same outcome in different ways. Inconsistency in reported outcomes causes well known problems for those who attempt to synthesise evidence, and many meta-analyses have to exclude key studies because relevant outcomes are not reported. Furthermore, the measured outcomes may not always be important to patients or health service users.

Much could be gained if an agreed core outcome set (COS) of a minimum number of appropriate and important outcomes was measured and reported in all clinical trials in a specific condition. Key stakeholders, including patients, should be involved in establishing COS, to ensure consideration of appropriate outcomes. COS may encompass all stages or severities of a condition or may focus on a particular disease category. Likewise, a COS may be for use in trials of all treatment types or only trials of a particular intervention. The scope of a COS should be defined to identify the relevant health condition, population and types of interventions.

The COMET Initiative (http://www.comet-initiative.org/) aims to foster and facilitate methodological research in the area of standardising outcomes, to develop much needed standards for methods of COS development and to develop and maintain a publically available internet-based resource to collate the knowledge base for COS development.

